# Experimental infection of carpet pythons (*Morelia spilota*) with sunshinevirus

**DOI:** 10.1128/jvi.01652-25

**Published:** 2026-03-03

**Authors:** Jane P. Wesson, Mark A. O'Dea, Andrew J. Currie, Cathy M. Shilton, Timothy H. Hyndman

**Affiliations:** 1 School of Veterinary Medicine, College of Environmental and Life Sciences, Murdoch University 5673, Murdoch, Western Australia, Australia; 2 Harry Butler Institute 5673, Murdoch, Western Australia, Australia; 3 School of Medical, Molecular, and Forensic Sciences, College of Environmental and Life Sciences, Murdoch University 5673, Murdoch, Western Australia, Australia; 4 Centre for Molecular Medicine and Innovative Therapeutics, Health Futures Institute, Murdoch University 5673, Murdoch, Western Australia, Australia; 5 Wesfarmers Centre of Vaccines and Infectious Diseases, The Kids Research Institute Australia, Perth, Australia; 6 Berrimah Veterinary Laboratories, Darwin, Australia; University of Kentucky College of Medicine, Lexington, Kentucky, USA

**Keywords:** RNA virus, experimental infection study, python

## Abstract

Sunshinevirus was originally isolated in 2008 from a collection of Australian pythons suffering an outbreak of neuro-respiratory disease. Sunshinevirus infection cases, including chronic asymptomatic infections, have subsequently been detected throughout Australia by PCR testing. To investigate the association between sunshinevirus infection and disease, controlled experimental infection studies in carpet pythons (*Morelia spilota*) were undertaken. The study aims were to determine if there was a causal relationship between sunshinevirus and disease, as well as collecting information on potential virus transmission routes, infectious doses, target organs for viral replication and pathology, and incubation period. Between August 2016 and February 2018, six pythons were inoculated with sunshinevirus, two were sham inoculated as control animals, and two were co-housed with infected animals. All animals were sampled weekly and sunshinevirus was detected in virus-inoculated animals by PCR 6–14 days post-inoculation. Five of the six virus-inoculated carpet pythons developed neuro-respiratory disease, and sunshinevirus could be re-isolated from liver and kidney tissues as well as blood. One of the co-housed pythons became infected, consistent with natural transmission. The other co-housed python remained uninfected. Clinical signs commenced 3–7 months after viral exposure. They were slow to develop, often subtle, and predominantly neurological. Histologically, there was a non-suppurative encephalomyelitis, with the most consistent finding being vacuolation and gliosis in the brain. Cytoplasmic inclusions were found in a range of predominantly epithelial tissues. Infected pythons shed infectious virus 2.5–7 months before they showed obvious clinical signs of disease and remained viremic (for several months) until euthanasia.

**IMPORTANCE:**

Australian pythons are extensively kept in captivity. Since the 1990s, native Australian snakes with neuro-respiratory signs of disease, sometimes fatal, had been presenting to veterinarians in Australia. Research conducted at Murdoch University on some of these snakes resulted in the discovery of a novel virus, now named sunshinevirus (Sunviridae). Widespread follow-up PCR testing of pythons throughout Australia linked sunshinevirus infection to both asymptomatic virus-carriers and pythons showing signs of disease. Experimental infections were conducted to determine whether sunshinevirus infection caused disease in Australian pythons. The experiments demonstrated a causal relationship by fulfilling Koch’s postulates, and 5/6 infected animals developed neurological disease. Infected pythons shed virus persistently for months, but control animals remained uninfected. Our findings provide veterinarians with valuable information for the management of sunshinevirus in zoos and private collections.

## INTRODUCTION

Sunshinevirus was first discovered in Australia, following an outbreak of neurological and respiratory disease in captive Australian pythons in 2008 (1). The first isolate, named Sunshine Coast virus (SunCV), belongs to the species *Sunshinevirus reptilis*, which is currently the only species in the family *Sunviridae* (genus *Sunshinevirus*; order *Mononegavirales*) (2). The virus has been detected in most Australian python species and from all Australian states and mainland territories except Western Australia (3). Outside of Australia, sunshinevirus has been identified by PCR in ball pythons (*Python regius*) from Germany (4) and in at least two captive red-tailed boa constrictors (*Boa constrictor constrictor*) from Thailand (5).

Although snakes may be infected asymptomatically, typical clinical signs of neurological disease include incoordination, mental dullness, diminished righting reflexes, opisthotonos, erratic mouth gaping, and head tremors (6, 7). Respiratory signs, such as dyspnea or mild oral discharge, are reported less commonly and are sometimes noted as being chronic or low-grade. Non-specific signs have been commonly reported and include lowered levels of consciousness, dysecdysis, anorexia, regurgitation, stomatitis, and sudden death. The longest documented period of viral shedding without clinical signs of disease is more than 2 years (7, 8).

Histopathology associated with infection has been described from a limited number of cases (6). Lesions were primarily found in the hind brain and were characterized by mild to marked vacuolation of white matter tracts with associated mild to moderate gliosis. Lymphocytic perivascular cuffing and meningeal infiltration were observed but were uncommon. Variable changes were found in the lungs. Inclusion bodies were uncommon, being seen only rarely in cells of the choroid plexus and renal tubules (6, 9). Histological examination of a ball python from Germany that was PCR-positive for sunshinevirus showed a mild multifocal lymphohistiocytic encephalitis (4). Two red-tailed boa constrictors in Thailand that were PCR-positive for sunshinevirus showed renal, hepatic, and pancreatic pathology (5). Prominent intracytoplasmic inclusions containing RNA were found in renal, hepatic, and pancreatic tissues in both boa constrictors. The brain tissues were not available for assessment.

In cases where outbreaks of disease associated with sunshinevirus have been described, a source of infection has not been identified. The virus may have been introduced in infected but asymptomatic new acquisitions (carriers). In one case, there were 12-month quarantine protocols in use (10). Vertical transmission of sunshinevirus has been demonstrated, with virus detected by PCR in dead embryos from a carpet python (*Morelia spilota*) clutch (10). However, no virus was detected from any of the live hatchlings from the same clutch, so this may be a dead-end method of transmission. Sunshinevirus has been detected by PCR in oral and cloacal swabs and blood (6, 10), indicating these fluids are potential sources of infection.

## MATERIALS AND METHODS

### Live animals

**Fig 1:**
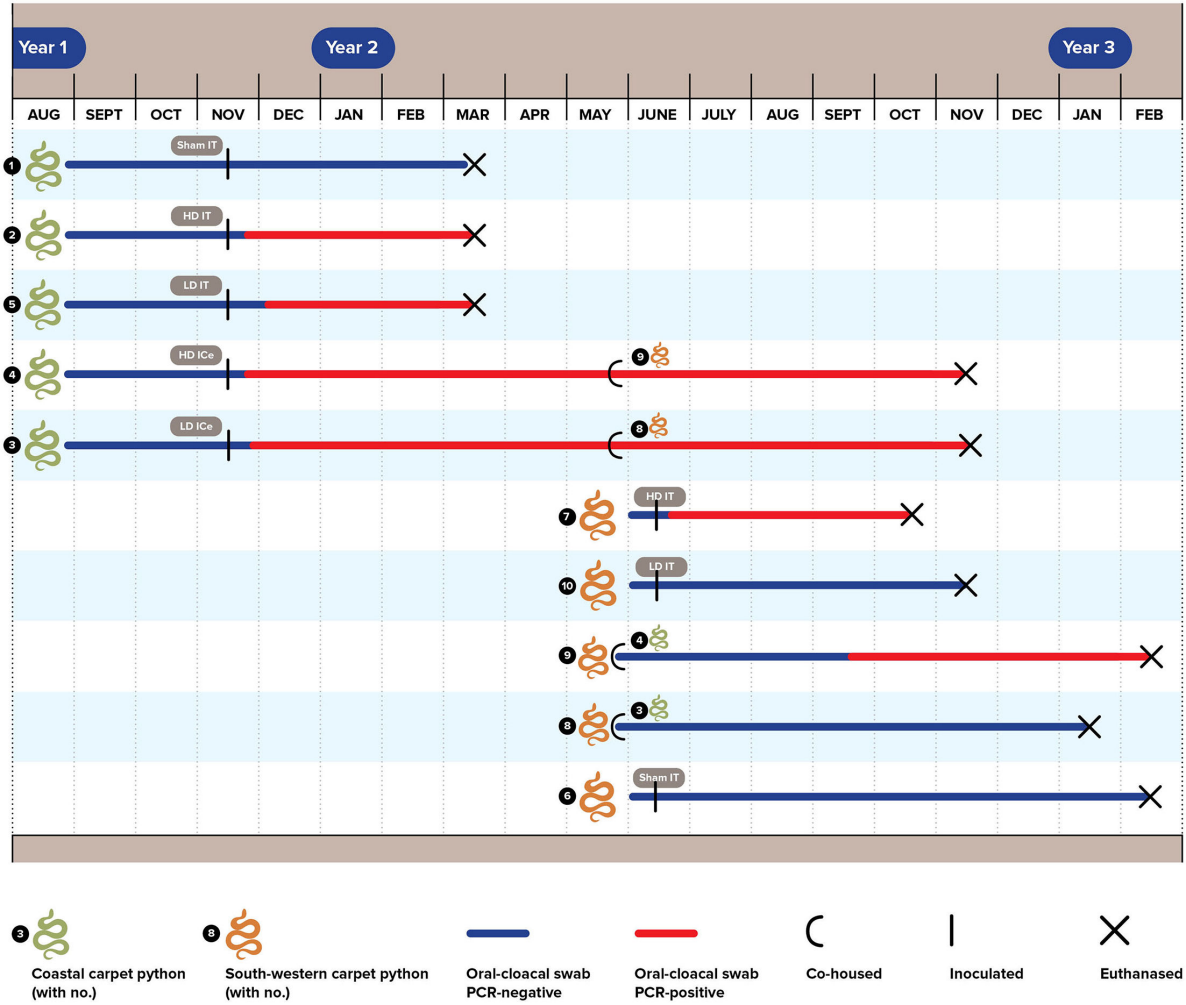
Sunshinevirus experimental infection study timeline. Five coastal carpet pythons (*Morelia spilota mcdowelli*, colored green in the figure) formed the first phase of the study. Five south-western carpet pythons (*Morelia spilota imbricata*, colored brown in the figure) joined the study in the second phase. Pythons 8 and 9 in the second phase were co-housed with infected pythons 3 and 4, respectively, from the first phase. Pythons 1 and 6 (the top and bottom snakes in the figure) were sham-inoculated controls. HD = high dose, ICe = intracoelomic, IT = intratracheal, LD = low-dose.

All animal work was conducted under permit from the Murdoch University Animal Ethics Committee (Permit Number R2792/15). The first phase of the study (Fig. 1) involved experimentation with different methods of administration and virus inoculation doses, to determine whether experimental infection was possible. Following successful infection of the inoculated snakes, phase two focused on characterizing the disease associated with viral infection.

Carpet pythons (*Morelia spilota*) were chosen for this study as they are the species most commonly diagnosed with sunshinevirus (3). Coastal carpet pythons (*M. s. mcdowelli*) were used in phase one of the study, and south-western carpet pythons (*M. s. imbricata*) were used in phase two of the study. All pythons went through a selection, testing, and acclimatization process to ensure that they were suitable for the study. Before inoculation, all pythons were tested for viruses that produce neurological and/or respiratory signs that could confound the results of this study. Combined oral-cloacal swabs from all pythons were tested twice by PCR for sunshinevirus (method below), reptarenaviruses (11), reptile bornaviruses (12), ferlaviruses (13, 14), snake serpentoviruses (nidoviruses) (method below), and reptile orthoreoviruses (15). All test results were negative. Blood films and fecal samples were examined for parasites (16), and none were detected.

The pythons were housed in a single room in a purpose-built animal research facility at Murdoch University, in Perth, Western Australia. They were housed in custom-built python enclosures with melamine-faced chipboard walls, a mesh ventilation panel in the back, and glass doors on the front. Each python had its own set of husbandry equipment, and all procedures (feeding, sampling) were conducted on the control animals before being carried out on virus-exposed pythons. Pythons were offered rodents weekly, size-matched to meet the python’s base metabolic rate energy requirements (17).

### Virus propagation and inoculation

Sunshine Coast virus (SunCV) was propagated in viper heart cells (VH2, ATCC CCL-140) in complete growth medium (cell medium [EMEM, Sigma-Aldrich], 2.5% or 5% fetal bovine serum [Serena, heat inactivated at 56°C for 30 min], 1% L-glutamine [200 mmol/L, Sigma-Aldrich], 0.2% Penicillin G/streptomycin [5,000 IU/mL penicillin and 5 mg/mL streptomycin, Sigma-Aldrich], 1% Amphotericin B [250 µg/mL, Sigma-Aldrich], and 0.05% enrofloxacin [Baytril oral solution 25mg/mL, Bayer]) in a humidified incubator with 5% CO_2_ at 30°C. For phase one, the study virus from the 10th passage was used as inoculum. Virus stocks were frozen at −20°C 3 weeks prior to inoculation. Pythons were inoculated intratracheally (*n* = 2) or intracoelomically (*n* = 2). Intracoelomic injection is an invasive procedure, used to bypass a number of natural defences, and, therefore, improves the likelihood of establishing an infection. This route can also mimic natural infection by hematophagous arthropods. The intratracheal route more closely mimics natural respiratory exposure. Viral inoculation doses were calculated and inocula made up with culture medium to the desired volume: 1 mL for snakes above 1.0 kg or 0.5 mL for snakes below this weight. Small volumes of virus stock were retained to calculate the tissue culture infectious doses (TCID_50_/mL) of the freeze-thawed inoculated virus. The TCID_50_/mL value was adjusted for the animal’s weight in kg, to give a dose expressed as TCID_50_/kg. The infectious doses were 10^5.8^ TCID_50_/kg (high dose) and 10^3.8^ TCID_50_/kg (low dose). Uninfected cell culture medium was used to sham-inoculate the control python. For the second phase of the study, two south-western pythons were co-housed with coastal carpet pythons that had been infected for 6 months. Virus of the 9th passage was used to inoculate two other pythons, in a repeat of the first phase of the experiment. The freeze-thawed inoculated virus produced tissue culture infectious doses calculated as 10^3.7^ TCID_50_/kg (high dose) and 10^1.7^ TCID_50_/kg (low dose).

### Monitoring and sample collection

Throughout the experiment, pythons were monitored and scored daily for general health using a standardized checklist that incorporated aspects of behavior such as activity, alertness, movement, posture, and breathing (see Supplementary material). A monthly score included assessment of fecal output, feeding behavior, and python weight. Pythons were sampled the day before inoculation (Day −1) or, if co-housed, just prior to introduction (on Day 0), and then twice a week for 2 months. Thereafter, they were sampled once a week until they were euthanased. Standardized oral-cloacal swabs and whole blood were collected on each sampling day. Swab tips (Citoswab, Citotest Labware Manufacturing Co., Ltd., China) were broken off into 3 mL of sterile phosphate-buffered saline (PBS) in a 5 mL sterile tube. Whole blood (up to 0.5 mL) was collected from the ventral tail vein, and blood films were made using standard wedge technique. Blood films were stained using a modified Wright Giemsa stain (Quick Dip, Thermo Fisher Scientific Australia Pty Ltd, Riverstone, NSW) and cover-slipped. Additional samples were collected opportunistically during the study. These included feces, voided urine, skin swabs, shed skins, and hemipenal plugs or semen plugs. All samples, except shed skins, were placed into sterile containers and stored at −20°C.

### PCRs

For PCR testing, RNA was extracted from samples using the Invitrogen PureLink Viral RNA/DNA Mini kit (for swabs) or the Qiagen QIAamp cador Pathogen Mini kit (catalog number 54,106, for blood and tissue samples), following the manufacturer’s recommendations. Tissues were pre-treated by organic extraction (using phenol and chloroform).

The snake serpentovirus (nidovirus) assay used to test pre-inoculation samples was an in-house developed probe-based qPCR, targeting a conserved section of the nidovirus genome located in ORF1a. Primer and probe sequences were derived from the genome of *Morelia viridis* nidovirus (NC_035465.1, GenBank) and are described in Table 1. Reactions were performed using AgPath-ID One-Step RT-PCR reagents (catalog number AM1005, ThermoFisher) in a Qiagen Rotor-Gene Q thermocycler. Briefly, 10 µL of 2× master mix, 0.8 µL of 25× enzyme mix, 0.8 µL of each of primers python nido F and R (final concentration 0.4 μmol/L), 0.24 µL of python nido probe (final concentration 0.12 μmol/L), and 2 μL of RNA extract were combined with PCR-grade water in a 20 μL reaction volume. Reaction conditions were as follows: 45°C for 10 min, 95°C for 10 min, followed by 45 cycles of 95°C for 15 s, and 60°C for 45 s. All samples were run in duplicate.

**TABLE 1:** Primer and probe sequences for the detection of snake serpentovirus ORF1a gene and sunshinevirus polymerase gene

Primer or probe name	Primer or probe sequence (5' → 3')
Python nido F primer	CGGCAAGGTACCTGACTGTT
Python nido R primer	AGACTCGCTTGCTGCTTGAT
Python nido probe	CAL Fluor orange560-AGCTTCGCAGTCGAGCACCACGACG-BHQ1
Sunshinevirus F primer	CCAGATGAGTATGATCCTACGTCA
Sunshinevirus R primer	CGCTTCCTGAGAAACAACTTAGC
Sunshinevirus Probe	FAM-TGTTACAAGATAAAGCTATTGCTCCA-BHQ1plus

Primer and probe sequences for an in-house developed sunshinevirus qPCR targeting a segment of the RNA-dependent RNA polymerase gene derived from the genome of Sunshine Coast Virus (NC_025345.1, GenBank) are also detailed in Table 1. The reagents and thermocycler used were the same as those used for the snake serpentovirus qPCR. Briefly, 12.5 µL of 2× master mix, 1.0 µL of 25× enzyme mix, 1.25 µL of each of primers F and R (final concentration 0.5 μmol/L), 0.375 µL of probe (final concentration 0.15 μmol/L), and 5 μL of RNA extract were combined with PCR-grade water in a 25 μL reaction volume. Reaction conditions were as follows: 45°C for 10 min, 95°C for 10 min, followed by 40 cycles of 95°C for 15 s and 55°C for 45 s. All samples were run in duplicate. This qPCR detected sunshinevirus that had been sequenced by next-generation sequencing (1) and was used to inoculate the study snakes, but the qPCR products from the subsequent live animal work were not sequenced.

Specificity of the sunshinevirus qPCR was confirmed against samples of reptile carboviruses (family *Bornaviridae*), snake serpentoviruses, reptarenaviruses, ferlaviruses, reptilian orthoreoviruses, and mycoplasmas (from Australian pythons).

Positive control material was assayed for sunshinevirus by digital PCR (dPCR) so that the quantified extraction could be used as a standard for the sunshinevirus qPCR. The extracted RNA was converted to cDNA using a SensiFAST cDNA synthesis kit (catalog number BIO-65053, Bioline), following the manufacturer’s recommendations. dPCR was performed using a Bio-Rad QX200 droplet digital PCR system. Results, expressed as viral copies per microliter, were used to calculate the viral concentration in the original extraction. Samples were interpreted as positive if the number of viral copies was >0/µL.

### Euthanasia samples

Pythons were euthanased using 10 mg/kg of alfaxalone (Alfaxan, Jurox) IV to effect, followed by 100 mg/kg of pentobarbitone sodium IC (Lethabarb, Virbac). One side of the brain was sampled fresh, and the remainder was fixed in 10% neutral buffered formalin. Sections of the vertebral column containing spinal cord were collected from each python at the levels of the neck, heart, liver, kidney, and vent. A wide range of other tissues (brain, trachea, thymus, thyroid, heart, lung, esophagus, stomach, liver, gallbladder, spleen, pancreas, adrenal glands, gonads, small intestine, large intestine, kidneys, cloaca, muscle, and skin) were collected at necropsy. A duplicate set of five tissues (brain, gonad, lung, liver, and kidney) was collected into virus transport medium (Medium 199 [Sigma-Aldrich], 0.5% bovine serum albumin, 0.2% penicillin G/streptomycin, and 1% amphotericin B [as for cell maintenance above]) for virus isolation. Tissues in formalin were kept at ambient temperature for an average of 3 days, before being trimmed into cassettes for paraffin embedding. Following formalin-fixation, vertebral segments and skulls were decalcified in 5% (vol/vol) nitric acid, before being sectioned for histology.

### Virus isolation

Virus isolation was attempted on tissues, swabs, and blood. Tissues and blood that were collected from study snakes at necropsy were tested. A selection of swab samples with high viral copy numbers (by qPCR), especially from Phase 1, was tested by virus isolation. Tissues for virus isolation were aseptically diced or processed in a bead beater for 2 min at 3,000 oscillations using 0.5 mL of 1.0 mm zirconia silica beads in 1 mL of virus isolation medium (EMEM medium, 5.00% heat-inactivated fetal bovine serum, 1.00% L-glutamine, 1.00% penicillin G/streptomycin, 2.00% Amphotericin B, 0.10% enrofloxacin [Baytril oral solution 25 mg/mL, Bayer]). After mechanical dissection, the tissue was centrifuged at 2,000 *× g* for 10 min at 4°C. The supernatant was applied to VH2 cells for 1 h at room temperature. Cells were then rinsed with PBS, and virus isolation medium was applied. Cultures were passaged after an average of 7 days. Swabs were vortexed in sterile PBS and centrifuged as above before supernatant was retrieved and filtered through a 0.45 µm-syringe tip filter (Millex-HV syringe filter unit, Merck Millipore Ltd, Ireland). Filtered supernatant was applied to the VH2 cells as described above. Heparinized whole blood was serially diluted in sterile PBS (50% to 0.5% final concentration) and then applied to cells in the same way. Cytopathic effect was defined by the presence of syncytial cells and quantified as the number of quartiles affected. Supernatant from infected cultures was tested by qPCR to confirm sunshinevirus infection.

## RESULTS

### PCR and virus isolation

Sunshinevirus was first detected by PCR 6–14 days after inoculation in five of the six pythons that had been directly inoculated (Table 3). Both the oral-cloacal swab and blood samples were positive at that time, and all subsequent samples from these pythons were positive until they were euthanased. Python 10 was the only python inoculated with virus that was negative for sunshinevirus on all PCR tests of swabs and blood. Sunshineviral RNA was also detected in samples of urine, feces, and hemipenal plugs (or semen) of infected pythons and in skin swabs of pythons that were infected or were cohabiting with a python that was infected.

**Fig 2:**
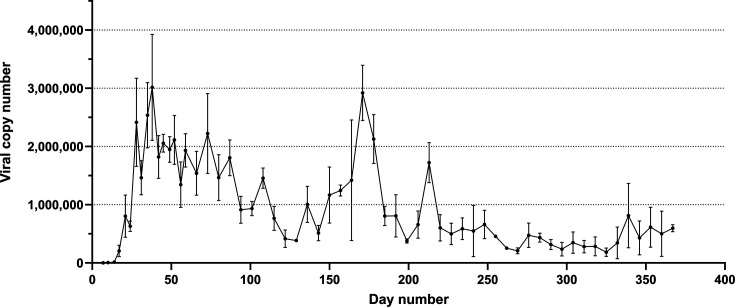
Mean viral RNA copies detected by qPCR from oral-cloacal swabs taken from sunshinevirus-inoculated Pythons 2 to 5. Error bars indicate the standard error of the mean. Days 0–124 *n* = 4; Days 126–367 *n* = 2.

The results from Pythons 2–5 were comparable. Following an initial sharp increase, the viral copy number detected in oral-cloacal swabs fluctuated during the study but did not fall below the limit of detection (Fig. 2). After Day 220, the viral copy numbers were generally lower. The results from Python 7 followed a similar trend, but with lower viral copy numbers detected in oral-cloacal swabs.

Pythons 8 and 9 were co-housed with infected pythons. Oral-cloacal swabs, but not blood, from these two snakes returned sporadic PCR-positive results during the first 72 days of cohabitation. Python 8 returned low-copy PCR-positive results (2–21 viral copies) from three samples (of the 10 collected) during this period. No further PCR-positive results were obtained for Python 8 (from swabs or blood) over 155 days of cohabitation with its infected cage-mate. Python 9 was PCR-positive on one occasion during the first 72 days of cohabitation, but this result was recorded as being equivocal, as a second test on this sample was negative. Swabs and blood from Python 9 then became persistently PCR-positive from Day 114 and Day 149, respectively.

**Fig 3:**
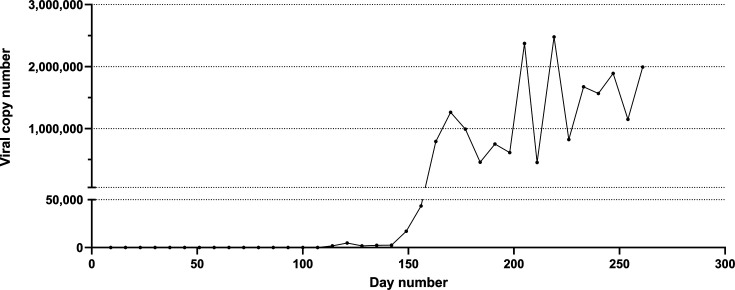
Viral copies detected by qPCR from oral-cloacal swabs taken from sunshinevirus-exposed Python 9 (co-housed with Python 4).

Python 9 was infected following cohabitation with Python 4. In contrast to the pythons that were inoculated with virus, there was a longer interval between the start of positive PCR results (Day 114) and the rapid increase in viral copy number (Fig. 3).

Sunshinevirus was isolated from selected weekly oral-cloacal swabs from all infected pythons. For directly inoculated pythons (Pythons 2–5 and 7), samples from Days 17–238 were used in virus isolation attempts. For the co-housed Python 9, samples from Days 198–205 were used in virus isolation attempts. No virus was isolated from swabs from sham-inoculated pythons (Pythons 1 and 6) or from Python 10, and no attempt was made to isolate virus from Python 8. Sunshinevirus was also isolated from tissues collected at necropsy. Virus was isolated from liver and kidney tissues, and blood, but not from brain, lung, or gonad (Table 2).

**TABLE 2:** Sunshinevirus isolated from experimentally-inoculated python tissues^*a*^

Python ID	Inoculation given^*b*^	Oral-cloacal swab PCR result	d.p.i. or d.p.e.^*c*^	Brain	Lung	Liver, kidney (combined or separate)^*d*^	Gonad	Blood
Coastal carpet pythons								
2	High-dose IT	**Positive**	124	Neg	Neg	**Pos**	Equiv	Neg
5	Low-dose IT	**Positive**	125	Neg	Neg	**Pos**	Neg	Neg
4	High-dose ICe	**Positive**	367	Neg	Neg	Lv Neg Kd Neg	Neg	Neg^*e*^
3	Low-dose ICe	**Positive**	369	Neg	Neg	**Lv Pos** **Kd Pos**	Neg	Pos
1	Sham IT	Negative	124	Neg	Neg	Neg	Neg	Neg
South-western carpet pythons								
7	High-dose IT	**Positive**	126	Neg	Neg	**Lv Pos** **Kd Pos**	Neg	Neg^*f*^
10	Low-dose IT	Negative	155	Neg	Neg	Lv Neg Kd Neg	Neg	Neg
9	Co-housed	**Positive**	262^*g*^	Neg	Neg	Lv Neg Kd Neg	Neg	n.t.
8	Co-housed	Negative	227	Neg	Neg	Lv Neg Kd Neg	Neg	Neg
6	Sham IT	Negative	244	Neg	Neg	Lv Neg Kd Neg	Neg	n.t.

^*a*^ Equiv = equivocal; Neg = negative; Pos = Positive (results in bold); n.t. not tested.

^*b*^ ICe = intracoelomic; IT = intratracheal.

^*c*^ d.p.i. = days post-inoculation; d.p.e. = days post-exposure.

^*d*^ Lv = liver; Kd = kidney.

^*e*^ on 3-day-old blood.

^*f*^ on frozen-thawed blood.

^*g*^ Python 9 was euthanased 262 days after having been placed with an infected python. At that time, it had been consistently positive on PCR tests for 148 days.

### Clinical signs

Onset of overt clinical signs occurred 2.5–7 months after infection was first detected by PCR (3–7 months after viral inoculation or 5–6 months after co-housing commenced) (Table 3). Subtle or non-specific signs, such as a head tremor or twitch, were occasionally seen earlier; the average onset of any observed clinical signs was 51 days (standard deviation = 40 days) for directly-inoculated snakes. In most cases, signs of disease were progressive. Neurological signs were most observed. Infection was not associated with weight changes.

**TABLE 3:** Virus detection and clinical signs seen in pythons experimentally exposed to sunshinevirus

	Python ID							
	2	5	4	3	7	10	9	8
Inoculation given or exposure^*a*^	HD IT	LD IT	HD ICe	LD ICe	HD IT	LD IT	Co-housed with 4	Co-housed with 3
Oral-cloacal swab PCR result^*b*^ (d.p.i.)^*c*^	**Pos.** **7­–124**	**Pos.** **14–125**	**Pos.** **7–367**	**Pos.** **10–369**	**Pos.** **6–­126**	Neg. 0­–155	**Pos. 1** **14** ^**c**^ **–262**	Neg. 0^c^–227
Total number of days that clinical signs were observed (first–last day that clinical signs were observed) (d.p.i.^*d*^)	2 (9–89)	22 (37–101)	20 (13–367)	183 (89–369)	14 (107–126)	0 (n.a.)	42 (163–262)	1 (134)
Clinical sign^*e*^								
Reluctance to move	+		+	++			+	
Disorientation				+	+		+	
Incoordination				+			+	
Convulsive head movement and mouth gaping					+++		++	
Head tremor or twitch	+	+	+				+	+
Muscle fasciculations			+	+				
Caudal flaccidity		+		+				
Tail kinking			+				+	
Intermittent raspy breathing			+					
Clear fluid in mouth				+				
Dysecdysis		++					+	
Anorexia				+	+		+	
Tenesmus							++	

^*a*^ HD = high dose, ICe = intracoelomic, IT = intratracheal, LD = low dose.

^*b*^ Pos. = positive (results in bold), Neg. = negative. All PCR-positive animals remained PCR-positive until euthanasia.

^*c*^ Python 9 returned a single PCR-positive result prior to Day 114, but this result was recorded as equivocal since retesting of this sample was negative. Similarly, Python 8 returned three PCR-positive results during the first 72 days of cohabitation, but these results were non-consecutive and low viral copy number, and no further positives were obtained from this python.

^*d*^ d.p.i. = days post-inoculation (or post-exposure to infected cage mate). The range is the days that the result extended for. n.a.= not applicable.

^*e*^ Clinical signs were subjectively assessed as mild (+), moderate (++), or severe (+++).

Neurological signs were observed in all six infected pythons and ranged from disorientation and incoordination to convulsive neck twisting and abnormal mouth gaping, which could be triggered by stimulus. The most common neurological sign was a tremor of the head, particularly while the python was in motion (intention tremor). In the more severely affected pythons, the head tremor was sometimes followed by exaggerated convulsive head movements associated with mouth gaping. Disorientation was observed in the more severely affected pythons and was particularly evident following a convulsive movement episode. Neuromuscular signs (five pythons) included abnormalities of the caudal musculature, either occasional kinking of the tail or hypotonia, and intermittent muscle fasciculations. One of the inoculated animals (Python 7) was euthanased on welfare grounds due to the severity of its neurological signs. The remaining pythons were electively euthanased when ongoing infection was achieved (Phase 1) or when the infection status of the pythons remained unchanged for weeks to months (Phase 2).

Non-neurological signs of disease included dysecdysis (two pythons), anorexia (three pythons), and dyspnea (one python). As disease progressed, the pythons had increasing difficulty in striking and prehending. Four pythons were noted as showing a reluctance to move. In three of these cases (Pythons 3, 4, and 9), this sluggishness was progressive and was associated with other signs of disease. None of the clinical signs were observed in either of the control pythons (Pythons 1 and 6). Python 8 was observed to have a head twitch on one occasion. This animal was co-housed with an infected python but was PCR-negative.

### Pathology

Significant pathology was found only in the pythons that were PCR-positive. There were no obvious differences in the pathological changes detected in the two carpet python subspecies; therefore, all cases have been combined for analysis. Python 9 (infected following co-housing with an infected cage-mate) had small patches of adhered shed. Otherwise, gross pathology in all snakes was unremarkable.

**Fig 4:**
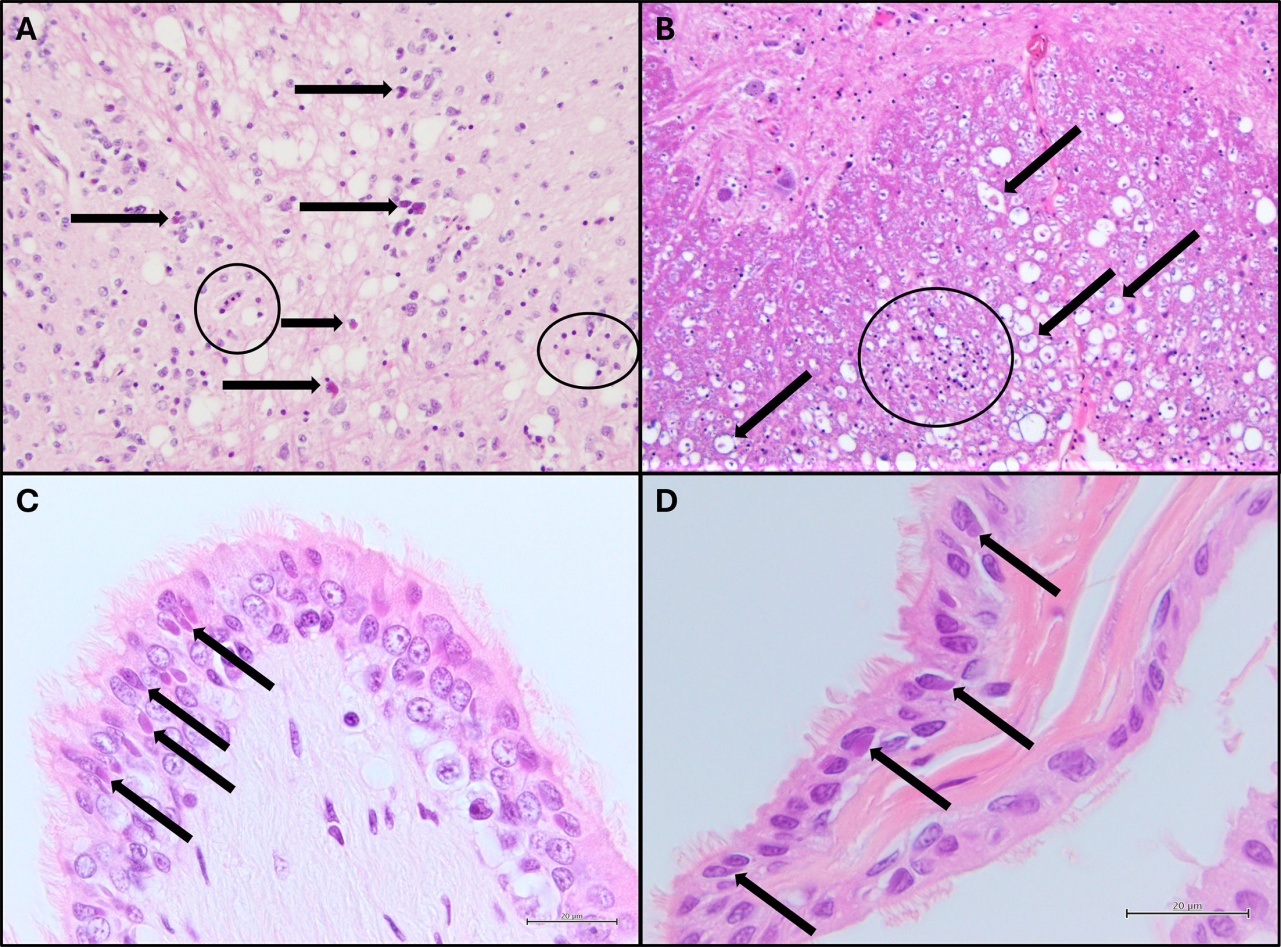
Histopathology in sunshinevirus-infected pythons (hematoxylin and eosin stain). (**A**) Moderate vacuolation of mid-brain white matter with amphophilic inclusions in glial cells (arrows). Glial cells are abnormally abundant (gliosis), and visible as small dark purple dots (two examples have been circled) (Python 7). (**B**) Severely vacuolated spinal cord white matter with moderate gliosis (circled) and digestion chambers (Wallerian degeneration) (arrows) (Python 9). (**C** and **D**). Amphophilic intracytoplasmic inclusions (arrows) in respiratory epithelium (Python 2). **C** is lung luminal epithelium and **D** is tracheal epithelium.

**Fig 5:**
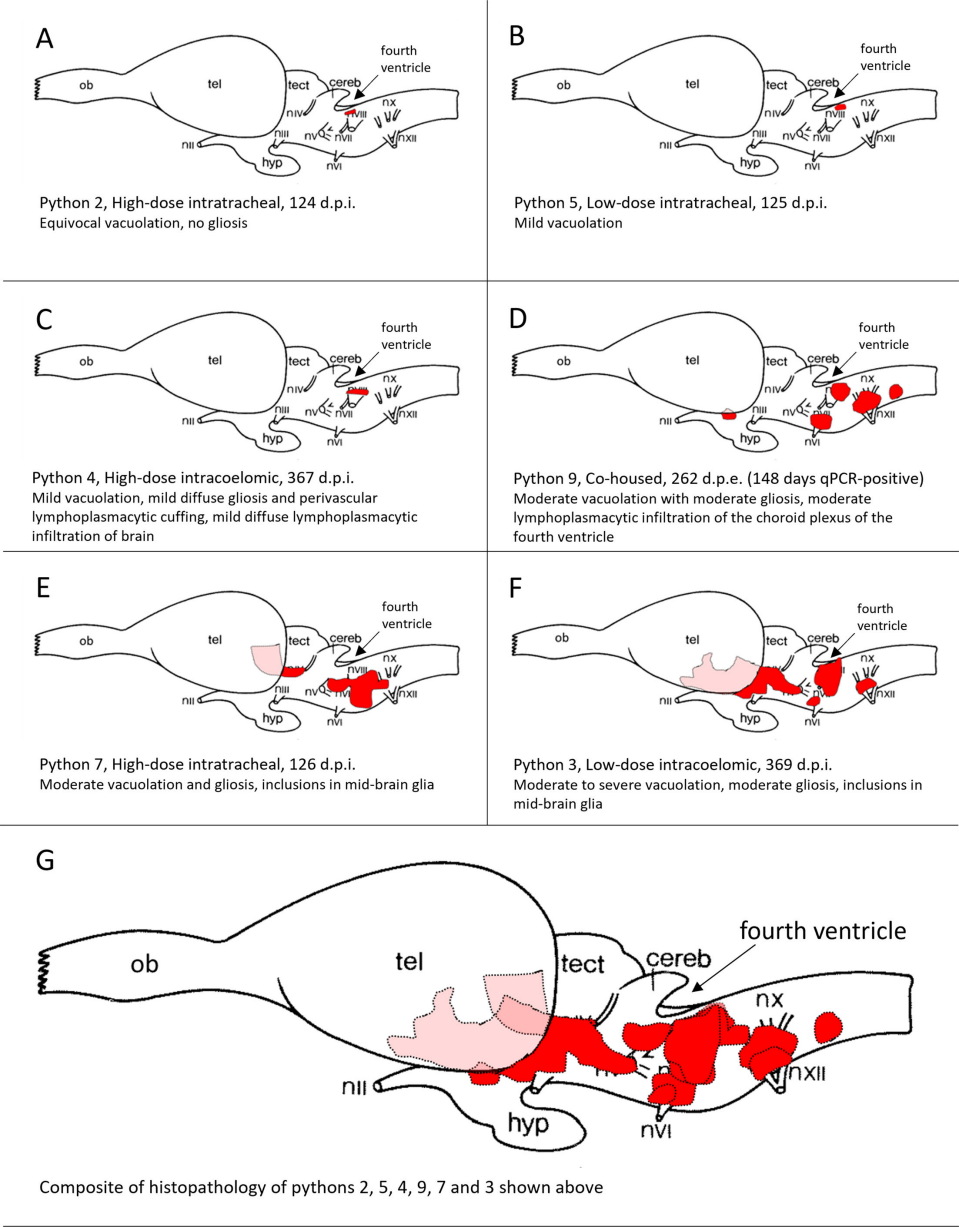
Location of vacuolation (shown in red) in sagittal sections of brains of pythons experimentally infected with sunshinevirus least affected (**A**) to most affected (**F**). **G** is a composite of the affected areas mapped in sections **A** to **F**. Light red areas are in the mid-brain, deep to the telencephalon. ob = olfactory bulb, tel = telencephalon, tect = tectum, cereb = cerebellum, hyp = hypophysis nii to nxii = cranial nerves II to XII d.p.i. = days post-inoculation, d.p.e. = days post-exposure.

Two broad groups of histological lesions were identified: neurological lesions and intracytoplasmic inclusions (Table 4). There was considerable variation in the severity of the lesions observed within tissues and between animals. However, pythons that had been infected longer had pathological changes in a greater range of tissues. The most consistent site for lesions was the white matter of the hindbrain, with the meninges and spinal cord sometimes also affected (Fig. 4 and 5). All pythons had brainstem pathology, with half (3/6) being assessed as moderate or severe and half (3/6) as mild. Spinal cord pathology was observed in 3/6 infected pythons. Amphophilic intracytoplasmic inclusions were observed in 5/6 (83%) infected pythons, most commonly in epithelial tissues. The respiratory epithelia were the most common sites for inclusions. Inclusions in the respiratory epithelia were assessed as severe in 2/5 (40%) cases, and in 3/5 (60%) cases they were assessed as mild. There was no clear relationship between the abundance of inclusion bodies seen and the length of infection.

**TABLE 4:** Neurological lesions and intracytoplasmic inclusions in sunshinevirus-inoculated or exposed pythons

Python ID (weight)^*a*^	Inoculation given or exposure^*b*^	d.p.i. PCR result^*c*^	Neurological lesions	Tissues containing intracytoplasmic inclusion bodies^*d*^
2 (2,110 g)	High-dose IT	124 +	Mild vacuolation of dorsal brainstem ventral to fourth ventricle (equivocal); mild regional lymphocytic infiltration of spinal meninges	Pulmonary LRE (+++); tracheal epithelium (+) (Fig. 4C and D)
5 (5,354 g)	Low-dose IT	125 +	Mild vacuolation of dorsal brainstem parenchyma immediately caudal to cerebellum; moderate gitter cell infiltration of fourth ventricle ependyma; moderate diffuse vacuolation of spinal white matter	Pulmonary LRE (+)
7 (190 g)	High-dose IT	126 +	Moderate vacuolation and associated gliosis primarily involving the white matter of the hind brain and mid-brain (Fig. 4A)	Pulmonary LRE (+); mid-brain glia (+) (Fig. 4A)
9 (1,156 g)	Co-housed	262^*e*^ +	Moderate vacuolation in mid-brain and hind brain with associated moderate gliosis and scattered necrotic neurons; moderate lymphoplasmacytic infiltration of the choroid plexus of the fourth ventricle; severe diffuse vacuolation of spinal white matter with associated digestion chambers (Wallerian degeneration) and moderate gliosis (Fig. 4B)	Pancreas (equivocal), oviduct epithelium (distal) (+)
4 (1,530 g)	High-dose ICe	367 +	Mild focal vacuolation of hind brain white matter; mild diffuse gliosis and perivascular lymphoplasmacytic cuffing; mild diffuse lymphoplasmacytic infiltration of brain and spinal meninges. Mild lymphoplasmacytic infiltration of a paravertebral ganglion	Pulmonary LRE (+); nasal epithelium (+)
3 (3,016 g)	Low-dose ICe	369 +	Vacuolation of white and gray matter, severe in mid- brain, moderate in hind brain; moderate gliosis; gitter cells perivascularly and in meninges; mild, patchy vacuolation of ventral spinal white matter	Mid-brain neurons, glia, and ependyma (++); pulmonary LRE (++); tracheal epithelium (+); gall bladder epithelium (+++); renal distal tubules and collecting duct epithelia (++)
10 (272 g)	Low-dose IT	155 −	None	None
8 (3,510 g)	Co-housed	227 −	None	None

^*a*^ Weight is in grams at euthanasia.

^*b*^ ICe = intracoelomic; IT = intratracheal.

^*c*^ d.p.i. = days post-inoculation, when euthanased; the PCR result is from testing of oral-cloacal swabs and indicates whether the python became infected.

^*d*^ The number of intracytoplasmic inclusion bodies was subjectively assessed as abundant (+++), moderate (++), or few (+). Inclusions were judged as “equivocal” when they were ill-defined or rare. LRE = luminal respiratory epithelium.

^*e*^ Python 9 was euthanased 262 days after being cohoused with an infected cage-mate. At that time, Python 9 had been consistently positive on PCR tests for 148 days.

Central nervous system (CNS) lesions were found in the six infected pythons. The most common neurological lesions were vacuolation of the white matter in the brain with accompanying gliosis and occasional evidence of Wallerian degeneration (Fig. 4A and B). Similar vacuolation of the spinal cord white matter was also observed in three pythons (Fig. 4B). Each of the five sections of spinal cord collected from an individual python (from cervical to vent-level) showed a similar level of vacuolation. Other significant findings in brain tissues were intracytoplasmic inclusions in the neurons, glia, or ependyma in two of the six pythons (Fig. 4A). Inclusions were found in tissues from all six infected pythons (Table 4, Fig. 4A, C and D). Amphophilic intracytoplasmic inclusions were found in various tissues, but most commonly in epithelia, particularly the mucosal epithelia of the respiratory tract (Fig. 4C and D).

Histological lesions were found most consistently in the brainstem. When brain tissues from the experimentally infected pythons are assessed—in order from least affected to most affected (Fig. 5)—a possible progression of the pathological changes in the brain associated with sunshinevirus infection emerges. The first indication of pathological change is seen around the fourth ventricle, with later changes extending to the mid-brain.

Many of the other histological findings were considered incidental. All pythons had some degree of hepatic lipidosis. Degenerative joint disease was noted in three large pythons (Pythons 3, 5, and 8). These same three pythons, as well as Python 10, exhibited varying degrees of renal glomerulosclerosis. Other general findings included aged granulomas, in the spleen (two pythons), lung (one python), or stomach (one python).

## DISCUSSION

Despite widespread detection by PCR of sunshinevirus in Australian pythons, prior to this study, its role in disease had not been established. This experimental infection study proves a causal relationship between Sunshine Coast virus and neuro-respiratory disease in Australian pythons by fulfillment of Koch’s postulates. Only pythons exposed to sunshinevirus developed infection and disease, while the unexposed pythons, as well as one python inoculated with a low dose of virus, remained uninfected. Infectious virus was recovered from the infected pythons. The principal target organ was the brain, with a wide range of epithelial tissues also affected.

The time from inoculation to a PCR-positive result (4–14 days) is consistent with the time interval reported in other experimental infection studies using viruses in snakes (18–35). Regardless of the virus type or the method of exposure, all studies that tested within 14 days of exposure to a virus reported viral infection within that time frame. In this study, intratracheal and intracoelomic inoculation produced infection more consistently and more rapidly than exposure through cohabitation, as has been reported in previous reptile virus infection studies (36, 37). It is possible that the dose of virus inoculated was much greater than a python would be exposed to under natural conditions. Also, inoculation into the trachea or coelom bypasses immune defenses such as antibodies in the upper respiratory tract (38) or the skin barrier (39).

The results of the present study provide some insight into the minimum dose of sunshinevirus that is needed to establish an infection (the minimum infectious dose), as not all inoculated pythons became infected. An inoculation dose (independent of animal bodyweight) of 10^1.0^ TCID_50_ did not result in infection, whereas all doses equal to or greater than 10^3.0^ TCID_50_ did result in infection. This is comparable to the infectious doses described for a range of other viruses used in reptile virus experimental infection studies (35, 37, 40–51).

Since a virus was cultured from oral-cloacal swab solutions that contain only small traces of sampled material, it is likely that there was sufficient virus being shed via the mouth and/or cloaca to infect another snake. However, the transfer of infection from Python 4 to Python 9 did not occur until after approximately 4 months of cohousing, and the transfer of infection from Python 3 to Python 8 did not occur during six months of cohabitation. The control pythons, which were housed directly adjacent to infected animals and shared a common airspace for more than a year, were negative on all tests. Airborne transmission of virus depends on many factors, including the presence of animals shedding virus, temperature, humidity, and stocking density (52). The high husbandry standards employed (climate-controlled room, filtered air, and removing any urine or feces within 24 h with spot-cleaning) may have influenced these results. Further investigations are required on the routes of natural acquisition of virus and on the possibility of fomites (especially those carrying body fluids or wastes) being a means of horizontal transmission.

The incubation period for sunshinevirus is likely to be many months. Clinical signs were slow to develop, often subtle, and were usually neurological or non-specific. In most infected pythons, clinical signs of disease were progressive. Obvious signs of disease were not seen until 3−7 months after inoculation or exposure to the virus, which was 2.5−7 months after infection was first detected by PCR. The infectious period lasted many months and pythons were shedding infectious virus long before they showed obvious clinical signs of disease. Most of the inoculated pythons were shedding infectious virus from at least Day 17. Since a virus was isolated from blood and tissues at necropsy, the pythons in this experiment likely remained infectious until they were euthanased (which ranged from Day 124 to Day 369).

Pathology was most consistently present in the CNS, matching previous observations of sunshinevirus-infected pythons (6). Infectious agents are known to enter the CNS in two main ways. The most common method of entry is via a hematogenous route after multiplying in other host tissues (53), culminating in penetration of either the blood-brain barrier or the blood-cerebrospinal fluid (CSF) barrier. The second method is via a cranial nerve (CN), particularly CN I (olfactory and vomeronasal), which can provide a pathway for pathogens directly to the brain (54, 55, 56). A hematogenous route for sunshinevirus infection of the CNS is supported by two forms of evidence from this study. First, an infectious virus was detected in the blood, as well as vascular tissues such as the liver and kidney, and pythons that had higher concentrations of viral RNA in the blood (Pythons 3, 7, and 9) correlated with the pythons that showed the most severe clinical signs of disease. Second, the histopathological changes in the brain (Fig. 5) suggest that CNS lesions first appear in the hind brain around the fourth ventricle, indicating the ventricular system as a possible point of entry to the brain. Inclusion bodies and lymphoplasmacytic infiltration observed in the choroid plexus and ventricular epithelium are consistent with this proposition. A similar lesion distribution is seen in canine distemper virus infections in the brain (57), where spread is via infected macrophages from the choroid plexus into the CSF. The virus then infects ependymal cells of the ventricular system, before spreading locally. There is limited evidence from this study supporting the entry of sunshinevirus to the brain via cranial nerves. The distribution of lesions in the brain shows some concentration around the origins of the cranial nerves, especially CNs VI, VIII, and XII. However, these three cranial nerves do not have neurons that are consistently in direct contact with the external environment (in contrast to CN I).

The CNS pathology associated with sunshinevirus infection overlaps that seen in other snake virus infections. While ferlavirus can produce both respiratory and neurological signs, the respiratory tract appears to be the main target (58). CNS pathology associated with ferlavirus infection in snakes is less commonly reported, and details have been found in only four reports (5, 59–61). Changes include demyelination and axonal swelling in the brain stem and proximal spinal cord, neuronal degeneration, gliosis, and perivascular cuffing in the hind brain, as well as occasional eosinophilic intracytoplasmic inclusion bodies in glial cells, neurons, or choroid plexus epithelium. Inclusion body disease, caused by reptarenaviruses, is a disease affecting snakes that is defined by a characteristic set of histological changes: variable non-suppurative encephalitis, plus specifically, an abundance of large, eosinophilic or amphophilic intracytoplasmic inclusion bodies in neurons in the CNS and/or epithelial cells from a variety of organs (62–64). Many of these CNS changes described for ferlavirus and reptarenaviruses are features that might also be seen in snakes infected with sunshinevirus. In Australia, ferlavirus has not yet been detected, and arenavirus has not been widely reported. Conversely, sunshinevirus has not been widely reported outside of Australia. However, the overlap in histopathology between these viruses demonstrates the importance of continuing to include them as differentials for a snake with neurological signs.

Conspicuous demyelination of the CNS is associated with relatively few viral infections (53, 57). These demyelinating diseases share characteristic morphology, immunopathological processes, glial responses, and evidence of early axonal degeneration with other demyelinating diseases, such as multiple sclerosis in humans (65). In these infections, the destruction of myelin occurs by a cell-mediated immunologic process. The pathological changes seen in the present study have the same morphology and cellular involvement as those described for these other demyelinating diseases. Ultrastructural examinations and immunological studies of inflammatory cytokines associated with neural support cells may provide additional insights on the processes occurring in sunshinevirus infections.

The experimentally infected pythons showed few signs of respiratory disease which has been reported previously in some cases of sunshinevirus infection (5, 6). Despite the lack of clinical signs of respiratory disease, histologically, the respiratory system was the second most common site for pathological changes. Amphophilic inclusion bodies were found in tissues of the respiratory system in five of the six infected pythons. Moderate lymphoplasmacytic infiltration was noted in the submucosa of multiple respiratory organs in two co-housed pythons (Pythons 4 and 9). Most pythons, whether infected or not, exhibited some degree of lymphoplasmacytic infiltration of the pulmonary luminal respiratory epithelium, indicating that this degree of infiltration in lung tissue may be normal, rather than a response to sunshinevirus infection. In contrast, ferlaviral infection is commonly associated with congestive or hemorrhagic proliferative pneumonia, often including interstitial inflammation and secondary bacterial infection, which may spread to other organs (34, 66–68).

The consistent observation of inclusion bodies in a range of tissues, and particularly in the respiratory epithelia, contrasts with previous reporting of sunshinevirus infection in Australia, where inclusion bodies were reported as rare (6). In contrast, in the two cases from Thailand (boa constrictors), inclusion bodies were found in the kidney, liver, and pancreas of both snakes (5). It is possible that the widespread finding of inclusion bodies in our study using Australian pythons is a result of the inoculation method or dose of virus. Other explanations for the greater prevalence of inclusion bodies noted in this experimental study include the length and severity of the viral infection and the wide range of tissues that were examined. For example, inclusion bodies were found in the epithelium of the gallbladder and oviduct, and these are tissues which may not be examined routinely.

Although the intracytoplasmic inclusions most likely represent viral material, the contents of these inclusions would require immunohistochemical and/or electron microscopic assessment to elucidate their composition. Inclusions were found in a range of predominantly epithelial tissues, but the greatest numbers were seen in respiratory epithelia in the inoculated pythons. This may suggest that the respiratory system is a site for viral replication (56, 58). However, no inclusions were seen in the respiratory epithelium of the python that became infected through cohabitation (Python 9), even after 5 months of continuous infection. An experimental infection study using reptarenaviruses in ball pythons (*Python regius*) and boa constrictors (*Boa constrictor*) (69) found inclusion bodies at 18 weeks post-inoculation in the boa constrictors, but not at 10 weeks post-inoculation in the ball pythons. The authors speculated that this might indicate that inclusion bodies accumulate slowly. In the present study, inclusions were most abundant in the respiratory epithelium of Python 2, which was the first python euthanased, at 124 days post-inoculation. Python 3, euthanased 369 days after inoculation, had inclusions in the widest range of tissues and was the python infected for the longest time.

Once established, infection persisted for many months, often asymptomatically, until termination. The establishment of persistent viral infection occurs when a primary infection is not cleared by the host immune response (70). There is no single mechanism by which this occurs. Contributing factors may include whether the virus is a cytopathic type, host cell types involved, and host immune responses. The predilection of sunshinevirus for the CNS could be a factor in establishing persistent infection since CNS tissues have reduced immune surveillance (53). However, since sunshinevirus is found in oral and cloacal secretions, infection must also occur elsewhere in the body. The epithelia of the respiratory tract, liver, kidneys, and reproductive tract could potentially produce the virus that is detected in swab samples. Beyond the evidence of gliosis in the CNS and speculation on demyelination being the result of a cell-mediated process, little is known of the host immune response to sunshinevirus infection. Further research is required on how sunshinevirus infection is established and the host response to it.

A limitation of experimental infection studies can be that it is not known how closely the experimental conditions—especially the routes of administration and doses of virus used—correlate with those occurring in naturally transmitted infections. There are several factors that suggest that the results from this study are comparable with results of naturally acquired infections. First, the study demonstrated infection acquired through close contact, rather than direct inoculation. Second, the patterns of infection and disease observed in all the experimentally infected animals were generally comparable, regardless of the route or dose of virus administered. Third, the clinical and pathological results were comparable with those observed in snakes that naturally acquired their infection.

The study cohorts were small (*n* = 10), and they included a range of sizes, both sexes, and two subspecies of *Morelia spilota*. It is not known whether the results would have been different if all the pythons had been more similar. Given that most exposed animals became infected, the variability of the animal cohort did not appear to be a barrier to infection. The use of viral inoculum of the 9th or 10th passage may have affected inoculum virulence, as a virus can show a reduction in virulence after it has been passaged many times (70).

Although viral copy numbers in the infected pythons fluctuated, they never fell below the limit of detection of the PCR, so a negative or positive PCR result will generally be a reliable indicator of an animal’s infection status. The exception will be during the period immediately following introduction of virus to a collection. This information can be used in collection biosecurity management. There is currently no specific treatment for sunshinevirus infection, and the prognosis for infected animals is poor, so preventing the virus from entering a collection should be the priority. It is, therefore, recommended that all new additions to a collection should be tested for sunshinevirus while in quarantine. Because of the persistence of infection, if a negative result is returned, it is recommended to test a second sample collected six weeks later. Positive results must be managed according to specific collection protocols. If enclosures that housed infected animals are to be reused, they should be thoroughly disinfected with 0.4% hypochlorite (10% household bleach) to destroy viral RNA. Otherwise, it has the potential to cause a misleading positive PCR result for an uninfected python introduced to the contaminated environment.

This study is the first experimental infection study using sunshinevirus, and the first published virus study using carpet pythons as the host species. Additional research examining the persistence of infection and its relation to the immune response will likely improve knowledge of progression of infection.

## Data Availability

Data reported in this paper are available from the corresponding author (Jane Wesson, j.wesson@murdoch.edu.au) upon reasonable request.

## SUPPLEMENTAL MATERIAL

Supplemental material jvi.01652-25-s0001.docx Snake monitoring sheet.

ASM does not own the copyrights to Supplemental Material that may be linked to, or accessed through, an article. The authors have granted ASM a non-exclusive, world-wide license to publish the Supplemental Material files. Please contact the corresponding author directly for reuse.

## References

[1] Hyndman TH, Marschang RE, Wellehan JFX, Nicholls PK. 2012. Isolation and molecular identification of Sunshine virus, a novel paramyxovirus found in Australian snakes. Infect Genet Evol 12:1436–1446. doi:10.1016/j.meegid.2012.04.022 22575820

[2] International committee on the taxonomy of viruses. 2022. Virus taxonomy: 2022 release, July 2022 ed. international committee on the taxonomy of viruses. Washington, USA

[3] Hyndman TH, Shilton C. 2018. An update on Australian reptile infectious diseases (2018). Proceedings of the 2018 Unusual Pet and Avian Veterinarians Conference.

[4] Marschang RE, Stöhr AC, Aqrawi T, Hyndman TH, Plenz B, Blahak S, Pees M. 2013. First detection of Sunshine virus in pythons (Python regius) in Europe. Proceedings of the Association of Reptilian and Amphibian Veterinarians Conference:15.

[5] Kongmakee P. 2014. Pathology and Molecular Diagnosis of Paramyxovirus Infection in Boidae and Pythonidae in Thailand. Master of Science in Veterinary Pathobiology. Chulalongkorn University, Bangkok, Thailand.

[6] Hyndman TH, Shilton CM, Doneley RJT, Nicholls PK. 2012. Sunshine virus in Australian pythons. Vet Microbiol 161:77–87. doi:10.1016/j.vetmic.2012.07.030 22883310

[7] Johnson R, Hyndman T. 2014. Sunshine and blisters. Proceedings of the Australian Veterinary Association Annual Conference. Perth, WA:H1.5.1-H1.5.2

[8] Hyndman TH, Shilton C. 2016. An update on Australian reptile viruses. Abstr Unusual Pet and Avian Veterinarians and Association of Avian Veterinarians Australasian Committee Joint Conference. Brisbane, Queensland

[9] Shilton CM, Hyndman T, Wesson J. 2019. Sunshinevirus, nidovirus and bornavirus in Australian snakes Wildlife Health and Pathology Short Course. Taronga Institute of Science and Learning. , Sydney, New South Wales

[10] Hyndman TH, Johnson RSP. 2015. Evidence for the vertical transmission of Sunshine virus. Vet Microbiol 175:179–184. doi:10.1016/j.vetmic.2014.11.008 25550284

[11] Stenglein MD, Jacobson ER, Chang L-W, Sanders C, Hawkins MG, Guzman DS-M, Drazenovich T, Dunker F, Kamaka EK, Fisher D, Reavill DR, Meola LF, Levens G, DeRisi JL. 2015. Widespread recombination, reassortment, and transmission of unbalanced compound viral genotypes in natural arenavirus infections. PLOS Pathog 11:e1004900. doi:10.1371/journal.ppat.1004900 25993603 PMC4438980

[12] Hyndman TH, Shilton CM, Stenglein MD, Wellehan JFX Jr. 2018. Divergent bornaviruses from Australian carpet pythons with neurological disease date the origin of extant Bornaviridae prior to the end-Cretaceous extinction. PLOS Pathog 14:e1006881. doi:10.1371/journal.ppat.1006881 29462190 PMC5834213

[13] Ahne W, Batts WN, Kurath G, Winton JR. 1999. Comparative sequence analyses of sixteen reptilian paramyxoviruses. Virus Res 63:65–74. doi:10.1016/s0168-1702(99)00059-3 10509717

[14] Kolesnik E, Hyndman TH, Müller E, Pees M, Marschang RE. 2019. Comparison of three different PCR protocols for the detection of ferlaviruses. BMC Vet Res 15:281. doi:10.1186/s12917-019-2028-0 31387580 PMC6685236

[15] Wellehan JFX, Childress AL, Marschang RE, Johnson AJ, Lamirande EW, Roberts JF, Vickers ML, Gaskin JM, Jacobson ER. 2009. Consensus nested PCR amplification and sequencing of diverse reptilian, avian, and mammalian orthoreoviruses. Vet Microbiol 133:34–42. doi:10.1016/j.vetmic.2008.06.011 18656318

[16] Wellehan JFX, Walden HDS. 2019. Chapter 32 - Parasitology (Including Hemoparasites), p 281–300. *In* Divers SJ, Stahl SJ (ed), Mader’s Reptile and Amphibian Medicine and Surgery, 3rd ed. W. B. Saunders, St. Louis, Missouri, USA.

[17] Calvert I. 2010. Nutrition, p 18–39. *In* Girling SJ, Raiti P (ed), BSAVA Manual of Reptiles, 2nd ed. British Small Animal Veterinary Association, Quedgeley, Gloucester, UK.

[18] Rosenbusch F. 1939. Equine encephalomyelitis in the Argentine in its experimental aspects. *In* Proc 6th Pacific Sci Congr 5:209-214

[19] Thomas LA, Eklund CM, Rush WA. 1958. Susceptibility of garter snakes (Thamnophis spp.) to western equine encephalomyelitis virus. Proc Soc Exp Biol Med 99:698–700. doi:10.3181/00379727-99-24468 13614471

[20] Karstad LH. 1961. Reptiles as possible reservoir hosts for eastern encephalitis virus. Trans N Am Wildl Nat Resour Conf:186–202.

[21] Gebhardt LP, Stanton GJ, Hill DW, Collett GC. 1964. Natural overwintering hosts of the virus of western equine encephalitis. N Engl J Med 271:172–177. doi:10.1056/NEJM196407232710402 14158354

[22] Gebhardt LP, Stanton GJ, de St. Jeor S. 1966. Transmission of WEE Virus to snakes by infected Culex tarsalis mosquitoes. Exp Biol Med (Maywood) 123:233–235. doi:10.3181/00379727-123-31452 5924436

[23] Hayes RO, Daniels JB, Maxfield HK, Wheeler RE. 1964. Field and laboratory studies on eastern encephalitis in warm- and cold-blooded vertebrates. Am J Trop Med Hyg 13:595–606. doi:10.4269/ajtmh.1964.13.595 14196060

[24] Spalatin J, Connell R, Burton AN, Gollop BJ. 1964. Western equine encephalitis in saskatchewan reptiles and amphibians, 1961-1963. Can J Comp Med Vet Sci 28:131–142. 17649511 PMC1494275

[25] Lee HW. 1968. Multiplication and antibody formation of Japanese encephalitis virus in snakes. Seoul J Med 9:157–161.

[26] Mifune K, Shichijo A, Ueda Y, Suenaga O, Miyagi I. 1969. Low susceptibility of common snakes in Japan to Japanese encephalitis virus. Trop Med 11:27–32.

[27] Thomas LA, Patzer ER, Cory JC, Coe JE. 1980. Antibody development in garter snakes (Thamnophis spp.) experimentally infected with western equine encephalitis virus. Am J Trop Med Hyg 29:112–117. doi:10.4269/ajtmh.1980.29.112 7352618

[28] Jacobson ER, Gaskin JM, Gardiner CH. 1985. Adenovirus-like infection in a boa constrictor. J Am Vet Med Assoc 187:1226–1227. doi:10.2460/javma.1985.187.11.1226 3001003

[29] Klenk K, Komar N. 2003. Poor replication of West Nile virus (New York 1999 strain) in three reptilian and one amphibian species. Am J Trop Med Hyg 69:260–262. 14628941

[30] Steinman A, Banet-Noach C, Simanov L, Grinfeld N, Aizenberg Z, Levi O, Lahav D, Malkinson M, Perk S, Shpigel NY. 2006. Experimental infection of common garter snakes (Thamnophis sirtalis) with West Nile virus. Vector Borne Zoonotic Dis 6:361–368. doi:10.1089/vbz.2006.6.361 17187570

[31] White G, Ottendorfer C, Graham S, Unnasch TR. 2011. Competency of reptiles and amphibians for eastern equine encephalitis virus. Am J Trop Med Hyg 85:421–425. doi:10.4269/ajtmh.2011.11-0006 21896798 PMC3163860

[32] Bosco-Lauth AM, Hartwig AE, Bowen RA. 2018. Reptiles and amphibians as potential reservoir hosts of chikungunya virus. Am J Trop Med Hyg 98:841–844. doi:10.4269/ajtmh.17-0730 29313469 PMC5930908

[33] Hoon-Hanks LL, Layton ML, Ossiboff RJ, Parker JSL, Dubovi EJ, Stenglein MD. 2018. Respiratory disease in ball pythons (Python regius) experimentally infected with ball python nidovirus. Virology (Auckl) 517:77–87. doi:10.1016/j.virol.2017.12.008 29329683

[34] Jacobson ER, Adams HP, Geisbert TW, Tucker SJ, Hall BJ, Homer BL. 1997. Pulmonary lesions in experimental ophidian paramyxovirus pneumonia of Aruba Island rattlesnakes, Crotalus unicolor. Vet Pathol 34:450–459. doi:10.1177/030098589703400509 9381656

[35] Pees M, Neul A, Müller K, Schmidt V, Truyen U, Leinecker N, Marschang RE. 2016. Virus distribution and detection in corn snakes (Pantherophis guttatus) after experimental infection with three different ferlavirus strains. Vet Microbiol 182:213–222. doi:10.1016/j.vetmic.2015.11.024 26711050

[36] Klenk K, Snow J, Morgan K, Bowen R, Stephens M, Foster F, Gordy P, Beckett S, Komar N, Gubler D, Bunning M. 2004. Alligators as West Nile virus amplifiers. Emerg Infect Dis 10:2150–2155. doi:10.3201/eid1012.040264 15663852 PMC3323409

[37] Maclaine A, Mashkour N, Scott J, Ariel E. 2018. Susceptibility of eastern water dragons Intellagama lesueurii lesueurii to Bohle iridovirus. Dis Aquat Org 127:97–105. doi:10.3354/dao03193 29384479

[38] Pastor LM. 1990. A morphological study of the tracheal epithelium of the snake Natrix maura. J Anat 172:47–57. 2272908 PMC1257202

[39] Tu MC, Lillywhite HB, Menon JG, Menon GK. 2002. Postnatal ecdysis establishes the permeability barrier in snake skin: new insights into barrier lipid structures. J Exp Biol 205:3019–3030. doi:10.1242/jeb.205.19.3019 12200405

[40] Clark HF, Karzon DT. 1972. Iguana virus, a herpes-like virus isolated from cultured cells of a lizard, Iguana iguana. Infect Immun 5:559–569. doi:10.1128/iai.5.4.559-569.1972 4344303 PMC422407

[41] Lamirande EW, Nichols DK, Owens JW, Gaskin JM, Jacobson ER. 1999. Isolation and experimental transmission of a reovirus pathogenic in ratsnakes (Elaphe species). Virus Res 63:135–141. doi:10.1016/s0168-1702(99)00067-2 10509725

[42] Origgi FC, Klein PA, Mathes K, Blahak S, Marschang RE, Tucker SJ, Jacobson ER. 2001. Enzyme-linked immunosorbent assay for detecting herpesvirus exposure in Mediterranean tortoises (spur-thighed tortoise [Testudo graeca] and Hermann’s tortoise [Testudo hermanni]). J Clin Microbiol 39:3156–3163. doi:10.1128/JCM.39.9.3156-3163.2001 11526144 PMC88312

[43] Origgi FC, Romero CH, Bloom DC, Klein PA, Gaskin JM, Tucker SJ, Jacobson ER. 2004. Experimental transmission of a herpesvirus in Greek tortoises (Testudo graeca). Vet Pathol 41:50–61. doi:10.1354/vp.41-1-50 14715968

[44] Johnson AJ, Pessier AP, Jacobson ER. 2007. Experimental transmission and induction of ranaviral disease in Western Ornate box turtles (Terrapene ornata ornata) and red-eared sliders (Trachemys scripta elegans). Vet Pathol 44:285–297. doi:10.1354/vp.44-3-285 17491069

[45] Schragen S. 2006. Experimetelle Infektion von juvenilen Boa constrictor mit einem Orthoreovirusisolat [Experimental infection of juvenile Boa constrictor with an orthoreovirus isolate]. Dr. med. vet, Justus Liebig University Giessen, Germany

[46] Allender MC, Mitchell MA, Torres T, Sekowska J, Driskell EA. 2013. Pathogenicity of frog virus 3-like virus in red-eared slider turtles (Trachemys scripta elegans) at two environmental temperatures. J Comp Pathol 149:356–367. doi:10.1016/j.jcpa.2013.01.007 23582975

[47] Darke S, Marschang RE, Hetzel U, Reinacher M. 2014. Experimental infection of Boa constrictor with an orthoreovirus isolated from a snake with inclusion body disease. J Zoo Wildl Med 45:433–436. doi:10.1638/2013-0194R.1 25000715

[48] Ariel E, Wirth W, Burgess G, Scott J, Owens L. 2015. Pathogenicity in six Australian reptile species following experimental inoculation with Bohle iridovirus. Dis Aquat Org 115:203–212. doi:10.3354/dao02889 26290505

[49] Hausmann JC, Wack AN, Allender MC, Cranfield MR, Murphy KJ, Barrett K, Romero JL, Wellehan JFX, Blum SA, Zink MC, Bronson E. 2015. Experimental challenge study of fv3-like ranavirus infection in previously FV3-like ranavirus infected eastern box turtles (Terrapene carolina carolina) to assess infection and survival. J Zoo Wildl Med 46:732–746. doi:10.1638/2015-0022.1 26667529

[50] Ariel E, Elliott E, Meddings JI, Miller J, Santos MB, Owens L. 2017. Serological survey of Australian native reptiles for exposure to ranavirus. Dis Aquat Org 126:173–183. doi:10.3354/dao03172 29160216

[51] Allender MC, Barthel AC, Rayl JM, Terio KA. 2018. Experimental transmission of frog virus 3-like ranavirus in juvenile chelonians at two temperatures. J Wildl Dis 54:716–725. doi:10.7589/2017-07-181 29878878

[52] Li X, Chai T, Wang Z, Song C, Cao H, Liu J, Zhang X, Wang W, Yao M, Miao Z. 2009. Occurrence and transmission of Newcastle disease virus aerosol originating from infected chickens under experimental conditions. Vet Microbiol 136:226–232. doi:10.1016/j.vetmic.2008.11.002 19091492

[53] Cantile C, Youssef S. 2016. Nervous System, p 250–406. *In* Maxie MG (ed), Jubb, Kennedy & Palmer’s Pathology of Domestic Animals, 6th ed. W.B. Saunders, St. Louis, Missouri.

[54] Kristensson K. 2011. Microbes’ roadmap to neurons. Nat Rev Neurosci 12:345–357. doi:10.1038/nrn3029 21587289

[55] Dando SJ, Mackay-Sim A, Norton R, Currie BJ, St John JA, Ekberg JAK, Batzloff M, Ulett GC, Beacham IR. 2014. Pathogens penetrating the central nervous system: infection pathways and the cellular and molecular mechanisms of invasion. Clin Microbiol Rev 27:691–726. doi:10.1128/CMR.00118-13 25278572 PMC4187632

[56] Burrell CJ, Howard CR, Murphy FA. 2017. Fenner and White’s Medical Virology. Fifth ed. Academic Press, Amsterdam.

[57] Miller AD, Zachary JF. 2017. Chapter 14 - Nervous System, p 805–907. In Zachary JF (ed), Pathologic basis of veterinary disease, 6th ed. Mosby.

[58] Rima B, Balkema-Buschmann A, Dundon WG, Duprex P, Easton A, Fouchier R, Kurath G, Lamb R, Lee B, Rota P, Wang L, ICTV Report Consortium. 2019. ICTV virus taxonomy profile: paramyxoviridae. J Gen Virol 100:1593–1594. doi:10.1099/jgv.0.001328 31609197 PMC7273325

[59] Jacobson E, Gaskin JM, Simpson CF, Terrell TG. 1980. Paramyxo-like virus infection in a rock rattlesnake. J Am Vet Med Assoc 177:796–799. doi:10.2460/javma.1980.177.09.796 7451314

[60] West G, Garner M, Raymond J, Latimer KS, Nordhausen R. 2001. Meningoencephalitis in a Boelen’s python (Morelia boeleni) associated with paramyxovirus infection. J Zoo Wildl Med 32:360–365. 12785686 10.1638/1042-7260(2001)032[0360:MIABSP]2.0.CO;2

[61] Sand MA, Latimer KS, Gregory CR, Rakich PM, Jacobson E, Pennick KE. 2004. Molecular diagnosis of paramyxovirus infection in snakes using reverse transcriptase-polymerase chain reaction and complementary deoxyribonucleic acid:ribonucleic acid in situ hybridization. J Vet Diagn Invest 16:442–448. doi:10.1177/104063870401600514 15460330

[62] Schumacher J, Jacobson ER, Homer BL, Gaskin JM. 1994. Inclusion body disease in boid snakes. J Zoo Wildl Med 25:511–524.

[63] Orós J, Tucker S, Jacobson ER. 1998. Inclusion body disease in two captive boas in the Canary islands. Vet Rec 143:283–285. doi:10.1136/vr.143.10.283 9787424

[64] Chang L-W, Jacobson ER. 2010. Inclusion body disease, a worldwide infectious disease of boid snakes: a review. Journal of Exotic Pet Medicine 19:216–225. doi:10.1053/j.jepm.2010.07.014

[65] Lempp C, Spitzbarth I, Puff C, Cana A, Kegler K, Techangamsuwan S, Baumgärtner W, Seehusen F. 2014. New aspects of the pathogenesis of canine distemper leukoencephalitis. Viruses 6:2571–2601. doi:10.3390/v6072571 24992230 PMC4113784

[66] Starck JM, Neul A, Schmidt V, Kolb T, Franz-Guess S, Balcecean D, Pees M. 2017. Morphology and morphometry of the lung in corn snakes (Pantherophis guttatus) infected with three different strains of ferlavirus. J Comp Pathol 156:419–435. doi:10.1016/j.jcpa.2017.02.001 28284556

[67] Homer BL, Sundberg JP, Gaskin JM, Schumacher J, Jacobson ER. 1995. Immunoperoxidase detection of ophidian paramyxovirus in snake lung using a polyclonal antibody. J Vet Diagn Invest 7:72–77. doi:10.1177/104063879500700111 7779968

[68] Woo PCY, Martelli P, Hui S-W, Lau CCY, Groff JM, Fan RYY, Lau SKP, Yuen K-Y. 2017. Anaconda paramyxovirus infection in an adult green anaconda after prolonged incubation: Pathological characterization and whole genome sequence analysis. Infect Genet Evol 51:239–244. doi:10.1016/j.meegid.2017.04.008 28404483

[69] Stenglein MD, Sanchez-Migallon Guzman D, Garcia VE, Layton ML, Hoon-Hanks LL, Boback SM, Keel MK, Drazenovich T, Hawkins MG, DeRisi JL. 2017. Differential disease susceptibilities in experimentally reptarenavirus-infected boa constrictors and ball pythons. J Virol 91:e00451-17. doi:10.1128/JVI.00451-17 28515291 PMC5651717

[70] Rall GF, Racaniello VR, Skalka AM, Flint J. 2015. Principles of Virology, Volume II: Pathogenesis & Control. ASM Press, Washington, DC.

