# Supplementary material for: Experimental infection of carpet pythons (Morelia spilota) with sunshinevirus
Source: J Virol. 2026 Mar 3;100(3):e01652-25. doi: 10.1128/jvi.01652-25 (PMC13011464; doi:10.1128/jvi.01652-25)
Supplement: Supplemental material — Snake monitoring sheet. [file jvi.01652-25-s0001.docx]

**SNAKE MONITORING SHEET**

**AEC Project No: ____**

| *Principal Investigator:*  *Co-Investigator =* ***contact:*** | | *Phone:*  *Email:* | | | | | | |
| --- | --- | --- | --- | --- | --- | --- | --- | --- |
| *Cage#/Animal ID* | | *Species/Sex/Age* | | | | | | |
| Frequency of monitoring: | | Daily | | | | | | |
| Day number |  | |  |  |  |  |  |  |
| Date (+/- time) |  | |  |  |  |  |  |  |
| Procedure |  | | Feed |  |  |  |  | Sample |
| **Daily or alternate days** |  | |  |  |  |  |  |  |
| **1. Activity/Alertness** *(Normal=0; altered routine=1; limited response to handling=2; moribund/unconscious=3)* |  | |  |  |  |  |  |  |
| **2. Movement/Posture** *(Normal=0; slight incoordination=1; tremor/flaccidity=2; star-gazing/paralysis=3)* |  | |  |  |  |  |  |  |
| **3. Breathing** *(normal=0; forks of tongue not separating=1; audible respiratory noises/nasal discharge=2; open mouthed breathing at rest=3)* |  | |  |  |  |  |  |  |
| **4. VAP site (if fitted)** *(normal=0; skin changes=1; swelling around site=2; discharge/ulceration=3)* |  | |  |  |  |  |  |  |
| **5. Injection site (if applicable)** *(normal=0; skin changes=1; swelling around site=2; infection=3)* |  | |  |  |  |  |  |  |
| **6. Hydration** *(normal=0; =1; skin tenting=2; skin tenting & sunken eyes=3)* |  | |  |  |  |  |  |  |
| **7. Skin** *(Normal=0; changes to skin=1; abnormal skin shedding=2; bleeding or infected wounds=3)* |  | |  |  |  |  |  |  |
| **8. Eyes** *(normal=0; change in clarity or colour=1; swelling/ discharge/squinty eye/retained spectacle=2; =3)* |  | |  |  |  |  |  |  |
| **Monthly** |  | |  |  |  |  |  |  |
| **9. Eating** *(normal=0; altered routine=1; refused last 2-3 feeds offered=2; refused last 4 or more feeds offered=3)* |  | |  |  |  |  |  |  |
| **10. Faeces and urates** *(normal=0; change in frequency or colour=1; constipation/discomfort=2; cloacal prolapse =3)* |  | |  |  |  |  |  |  |
| **11. Body weight** (g) *(normal weight & growth rate=0; reduced growth weight=1; weight loss>10% within a month =2; weight loss >10% within two weeks=3)* |  | |  |  |  |  |  |  |
| **Monitored by:** |  | |  |  |  |  |  |  |
| **Other Comments**  **UA=unable to assess** |  | |  |  |  |  |  |  |

**Actions and Interventions:**

**Veterinary assessment required**: A score of 2-3 for any criterion listed above.

**Criteria for euthanasia:** Assessment score of 3 for any of the following clinical observations: Activity/Alertness, Movement/Posture, Breathing.

Any unexpected deaths are to be reported to Animal Welfare Officer immediately.

**Daily score totals for criteria 1-8**

| Date (+/- time) |  |  |  |  |  |  |  |
| --- | --- | --- | --- | --- | --- | --- | --- |
| Total score |  |  |  |  |  |  |  |

**Daily total scores:**

| 0 | Routine monitoring |
| --- | --- |
| 1-5 | Monitor daily; recheck with handling as required |
| 6-10 | Veterinary assessment required; perform physical examination |
| >10 | Assess for euthanasia; ultimate assessment lies with independent veterinarian if possible |

**Monthly score totals for criteria 9-11**

| Date (+/- time) |  |  |  |  |  |  |  |
| --- | --- | --- | --- | --- | --- | --- | --- |
| Total score |  |  |  |  |  |  |  |

**Monthly total scores:**

| 0 | Routine monitoring |
| --- | --- |
| 1-2 | Monitor daily or weekly; recheck with handling as required |
| 3-5 | Veterinary assessment required; perform physical examination |
| >5 | Assess for euthanasia; ultimate assessment lies with independent veterinarian if possible |

Figure Supp Mat 1 Python monitoring sheet
